# Association of Systemic Inflammation with Dietary Intake, Nutrition Impact Symptoms, and Eating-Related Distress Among Patients with Advanced Cancer

**DOI:** 10.3390/healthcare12242533

**Published:** 2024-12-16

**Authors:** Koji Amano, Saori Koshimoto, Satomi Okamura, Tatsuma Sakaguchi, Sayaka Arakawa, Yoshinobu Matsuda, Akihiro Tokoro, Takashi Takeuchi, Eriko Satomi, Tamiki Wada, Makoto Wada, Tomomi Yamada, Naoharu Mori

**Affiliations:** 1 Department of Supportive and Palliative Care, Osaka International Cancer Institute, 3-1-69 Otemae, Chuo-ku, Osaka 541-8567, Japan; 2 Liaison Psychiatry and Psycho-Oncology Unit, Department of Psychiatry and Behavioral Sciences, Graduate School of Medical and Dental Sciences, Institute of Science Tokyo, 1-5-45 Yushima, Bunkyo-ku, Tokyo 113-8510, Japan; skoshimoto-rd@umin.ac.jp (S.K.); okaspsyc@tmd.ac.jp (T.T.); 3 Faculty of Human Nutrition, Department of Human Nutrition, Tokyo Kasei Gakuin University, 22 Sanban-cho, Chiyoda-ku, Tokyo 102-8341, Japan; 4 Department of Medical Innovation, Osaka University Hospital, 2-2 Yamadaoka, Suita 565-0871, Japan; satomi.okamura@dmi.med.osaka-u.ac.jp (S.O.); tomomi.yamada@dmi.med.osaka-u.ac.jp (T.Y.); 5 Department of Palliative and Supportive Medicine, Graduate School of Medicine, Aichi Medical University, 1-1 Yazakokarimata, Nagakute 480-1195, Japan; sakaguchi.tatsuma.430@mail.aichi-med-u.ac.jp (T.S.); nmori@aichi-med-u.ac.jp (N.M.); 6 Department of Palliative Medicine, National Cancer Center Hospital, 5-1-1 Tsukiji, Chuo-ku, Tokyo 104-0045, Japan; sarakawa@ncc.go.jp (S.A.); esatomi@ncc.go.jp (E.S.); 7 Department of Psychosomatic Internal Medicine and Supportive and Palliative Care Team, NHO Kinki Chuo Chest Medical Center, 1180 Nagasone-cho, Kita-ku, Sakai 591-8555, Japan; matsuda.yoshinobu.tx@mail.hosp.go.jp (Y.M.); tokoro.akihiro.qb@mail.hosp.go.jp (A.T.); 8 Department of Psychiatry, Osaka University Graduate School of Medicine, D3 2-2 Yamadaoka, Suita 565-0871, Japan; twada@psy.med.osaka-u.ac.jp; 9 Department of Psycho-Oncology, Osaka International Cancer Institute, 3-1-69 Otemae, Chuo-ku, Osaka 541-8567, Japan; makoto.wada@oici.jp

**Keywords:** cancer, cachexia, systemic inflammation, C-reactive protein, symptom, distress, quality of life

## Abstract

Background: Serum C-reactive protein (CRP) levels are correlated with patient outcomes in cancer. This study aimed to determine associations between the CRP level and the dietary intake, symptoms, and eating-related distress (ERD). Methods: We conducted a multicenter survey among advanced cancer patients. Information on patient characteristics was retrieved from the electronic medical records. Data on patient outcomes were obtained through the questionnaire. Patients were categorized into the low CRP group (<5 mg/dL) and the high CRP group (≥5 mg/dL). Comparisons were calculated using the Mann–Whitney U test or chi-squared test. To assess associations between CRP levels and ERD, multivariate logistic regression analysis was performed. Results: A total of 191 patients were enrolled and divided into the low CRP group (*n* = 117) and the high CRP group (n = 74). The high CRP group had a more reduced dietary intake (*p* = 0.002) and more severe appetite loss (*p* = 0.008). The total scores of the ERD questionnaire (both the long and short versions) were significantly higher in the high CRP group (*p* = 0.040 and 0.029). The high CRP group also had significantly higher risks for ERD, as assessed using the long and short versions of the questionnaire (odds ratio [OR] 2.13, 95% confidence interval [CI] 1.10–4.11; OR 2.06, 95% CI 1.05–4.05). Conclusions: High CRP levels were significantly associated with reduced dietary intake, appetite loss, and ERD. A serum CRP value of 5 mg/dL may be a useful indicator for initiating cancer cachexia care.

## 1. Introduction

Previous research suggests that tumor-immune system crosstalk generates pro-inflammatory cytokines, leading to systemic inflammation, and systemic inflammation is associated with the mechanisms responsible for cachexia in various advanced cancers [1,2,3,4,5,6]. A recent review also reported that inflammatory, metabolic, and neuro-modulatory drivers may initiate processes that ultimately converge on advanced cachexia [7]. Thus, multiple events in cancer cachexia and a variety of symptoms in advanced cancer appear to be caused by alterations in the central nervous system (CNS), induced by pro-inflammatory cytokines and systemic inflammation [8,9,10].

Recently published evidence-based clinical guidelines insist on the importance of multimodal care for people affected by cancer cachexia [11,12,13,14]. However, no standard care has been established to manage cancer cachexia or address problems caused by cancer cachexia, despite its high prevalence and negative impacts on the quality of life (QOL) in this population [11,12,13,14]. Therefore, we performed a literature review and identified nine components of multimodal care for cachectic patients with advanced cancer and their family caregivers: physical symptom management, psychological symptom management, providing evidence-based nutritional care and exercise, improving adherence to multimodal treatments, assisting patients and family caregivers with psychological adjustment, helping patients and family caregivers cope, providing evidence-based information about cancer cachexia to patients and family caregivers, educating patients and family caregivers about cancer cachexia, and initiating end-of-life discussions with patients and family caregivers [9,15]. Nevertheless, there is limited evidence on the appropriate timing of multimodal care initiation for patients and family caregivers [9,15]. Hence, we should know the best timing perceived by patients to initiate multimodal interventions provided by a multidisciplinary team for patients with cancer cachexia.

Notably, the serum level of C-reactive protein (CRP) has served as an indicator of systemic inflammation, which has been correlated with physical and psychological symptoms, physical function, and survival in patients with advanced cancer [16,17,18,19,20,21,22,23,24,25]. These findings imply that CRP levels are useful in identifying patients who need multimodal care for cancer cachexia. Therefore, this study aimed to determine the relationships between serum CRP levels, dietary intake, nutrition impact symptoms (NISs), and eating-related distress (ERD), which are commonly regarded as cachexia-related issues, among patients with advanced cancer, in order to explore CRP levels as a guide to starting multimodal care among cachectic patients with advanced cancer.

## 2. Participants and Methods

### 2.1. Sites and Participants

In this study, a multicenter survey was implemented using a self-reported questionnaire to determine when it was appropriate to initiate multimodal interventions provided by a multidisciplinary team for patients with advanced cancer. The survey was conducted in hospital palliative care teams and palliative care units at six designated cancer hospitals in Japan between November 2023 and June 2024.

All patients meeting the eligibility criteria were enrolled. The following inclusion criteria were established: (1) patients who were newly referred to palliative care, (2) patients who were aged ≥ 18 years, (3) patients who were diagnosed with locally advanced or metastatic cancer and hematologic neoplasms, (4) patients who were aware of the diagnosis of cancer, and (5) patients who could complete a self-administered questionnaire. Patients who were prohibited by their attending physicians from eating by mouth for medical reasons and patients who were found to be in serious psychological distress during an interview with a palliative care physician were excluded. Patients who refused to participate in the study were also excluded. 

### 2.2. Ethics Approval and Consent to Participate

This study was performed following the ethical standards outlined in the Helsinki Declaration and the ethical guidelines for medical and health research involving human subjects presented by the Ministry of Health, Labor, and Welfare in Japan [26]. This study was approved by the Institutional Review Board at Osaka University Hospital for all participating institutes (approval number 23226). Individual informed consent from participants is not required by Japanese law in a non-invasive observational trial, and thus the requirement for written or oral informed consent was waived based on national ethical guidelines. Once participants completed and returned the questionnaire, they were considered to have consented to participate in this study. Patients who disliked participating in this study were requested to return the questionnaire with ‘no participation’ indicated.

### 2.3. Measurements

#### 2.3.1. Patient Characteristics and Anthropometric Measurements

Data on patient characteristics, including Eastern Cooperative Oncology Group (ECOG) performance status [27], fluid retention status that can affect body weight (i.e., edema, pleural effusion, and ascites), and cancer treatment status (i.e., pre-chemotherapy, chemotherapy, and never treated/previous treatment), were collected. Patients who did not undergo cancer treatments owing to their poor condition and patients who ceased anticancer treatment were categorized as never treated/previous treatment. The most recent blood test results within one week prior to enrollment were used.

Patient anthropometric measurements (i.e., height [m], current body weight [kg], and previous body weight [kg]) were recorded. The body mass index (BMI) was calculated by dividing the body weight by the height squared. The weight loss rate (WLR) over 6 months was calculated as follows: (previous body weight − current body weight)/previous body weight × 100. Patients with a 6-month WLR > 5% or BMI < 20 kg/m^2^ + 6-month WLR > 2% were diagnosed with cachexia, according to the international diagnostic criteria on cancer cachexia [2]. 

#### 2.3.2. Dietary Intake

We evaluated the association of CRP levels with patients’ dietary intake using the Ingesta-Verbal/Visual Analog Scale (IVVAS), a 10-point scale for assessing dietary intake in patients with cancer. The participants estimated how they were eating on a scale from 0 to 10 (0 means “nothing at all” and 10 means “as usual”, with higher scores indicating better dietary intake). In medical oncology, a dietary intake score of 7 or less suggests that patients have a nutritional risk of weight loss [28,29]. 

#### 2.3.3. Nutrition Impact Symptoms

We proposed the following new definition: an NIS is a symptom that decreases a patient’s desire and ability to eat, interferes with necessary nutritional intake, and increases the risk of malnutrition, lean body mass loss, and decreased quality of life [30]. We evaluated the associations of CRP levels with 19 NISs, assessed using a numerical rating scale ranging from 0 to 10 (0: none, 1 to 3: mild, 4 to 6: moderate, 7 to 9: severe, 10: very severe), as one of the patient-centered outcomes recommended by a systematic review and clinical recommendations edited by the American Society for Parenteral and Enteral Nutrition [31]. We also examined the relationships between CRP levels and the symptom scales included in the European Organization for Research and Treatment of Cancer Core Quality of Life Questionnaire (EORTC QLQ-C30) [32]. 

#### 2.3.4. Eating-Related Distress

We determined the association of CRP levels with ERD, measured using the questionnaire for eating-related distress among patients with advanced cancer (QERD-P). The QERD-P long version contains 3 entries in each of the 7 domains, for a total of 21 entries, and each item is rated on a 7-point Likert scale. High scores indicate worse distress. The QERD-P short version consists of 7 entries, each of which contains a representative entry selected from each of the 7 domains [33]. 

#### 2.3.5. Quality of Life

Patients’ QOL was evaluated using the global health status of the EORTC QLQ-C30 [32]. 

### 2.4. Statistical Analysis

#### 2.4.1. Relationships Between Serum C-Reactive Protein Levels, Dietary Intake, Nutrition Impact Symptoms, Eating-Related Distress, and Quality of Life

Patients were categorized into 2 groups using a cutoff value for the CRP levels (low CRP group [<5 mg/dL] and high CRP group [≥5 mg/dL]), following the practical guidance for the assessment of inflammation, which suggests that CRP levels greater than 5 mg/dL indicate severe inflammation [34]. Patient characteristics and anthropometric measurement data were presented as proportions (%) for categorical variables or medians (interquartile range [IQR]) for continuous variables, as appropriate. Comparisons between groups were calculated using the Mann–Whitney U test or chi-squared test, as appropriate. 

#### 2.4.2. Association of Serum C-Reactive Protein Levels with Eating-Related Distress

To assess the association between CRP levels and the ERD experienced by patients, a multivariate logistic regression analysis was performed. As a dependent variable, the ERD scores were divided into two categories based on the median. A multivariate model was adjusted for the CRP levels (<5 and ≥5 mg/dL), age in years, sex (female and male), primary site of cancer (upper and lower gastrointestinal tracts + liver, biliary system, and pancreas [gastrointestinal], lung, and others), treatment status (pre-chemotherapy + never treated/previous treatment [non-chemotherapy] and chemotherapy), and ECOG performance status (PS) (0–1, 2, and 3–4). 

The results were considered significant with a *p*-value < 0.05. All analyses were performed using SAS software, version 9.4 (SAS Institute, Cary, NC, USA).

## 3. Results

**Figure 1:**
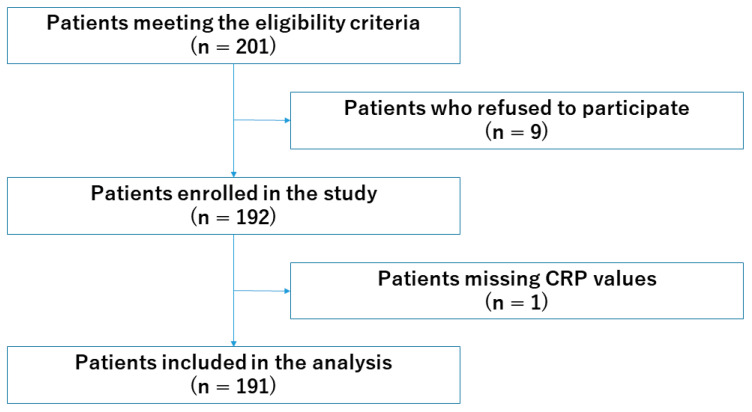
Flow diagram of patient participation in the study.

A total of 201 patients were eligible for inclusion in this study. Among them, 9 declined consent, and 1 was excluded due to a missing value regarding the serum CRP level. Thus, a total of 191 patients were enrolled in this study (Figure 1).

Patient characteristics according to serum CRP levels are summarized in Table 1. Among all patients, the median age was 67.0 years old. There were slightly more females (52.1%) than males (47.9%). The top three primary cancer sites were the lung (20.8%), liver, biliary system, and pancreas (17.2%); and upper and lower gastrointestinal tracts (12.5%). Moreover, 70.8% of the patients received anticancer treatment. The proportions of ECOG performance status 0–1, 2, 3, and 4 were 20.3%, 34.9%, 39.1%, and 5.7%, respectively. Furthermore, 64.1% of the patients had cancer cachexia or refractory cachexia. 

All patients were divided into the low CRP (n = 117) and high CRP (n = 74) groups. Significant differences were observed in ECOG PS (*p* = 0.045), weight loss rates (*p* = 0.043), fluid retention (*p* = 0.003), and serum albumin and CRP levels (both *p* < 0.001) between the two groups. Patients with high CRP levels had significantly more impaired performance status, higher weight loss rates, more severe fluid retention, and lower albumin levels than those with low CRP levels (Table 1). 

Relationships between the serum CRP levels and the dietary intake and NISs are shown in Table 2. The median dietary intake score of all patients assessed using the IVVAS was 5.0, and the median scores of the low CRP and high CRP groups were 5.0 and 4.0, respectively. The dietary intake of patients with high CRP levels was significantly lower than that of patients with low CRP levels (*p* = 0.002). Proportions of patients with a dietary intake score of 7 or less were 79.5% and 93.3% in the low CRP and high CRP groups, respectively. A significant difference was observed between the groups (*p* = 0.009). The high CRP group exhibited similar or stronger symptoms than the low CRP group, except for pain other than oral pain and shortness of breath. Significant differences were observed between the two groups in terms of vomiting and drowsiness (*p* = 0.012 and 0.041, respectively). Furthermore, the high CRP group had a significantly higher score of appetite loss, as included in the symptom scales of the EORTC QLQ-C30, than the low CRP group (the medians [IQR], 66.7 [33.3–100.0] and 66.7 [33.3–100.0]; *p* = 0.008) (Table 3). The breakdowns of score of appetite loss (0, 33.3, 66.7, 100) were 16 (15.5%), 28 (27.2%), 31 (30.1%), 28 (27.2%) in the low CRP group and 4 (5.6%), 19 (26.8%), 13 (18.3%), 35 (49.3%) in the high CRP group, respectively. 

Relationships between the serum CRP levels and ERD are summarized in Table 4. The high CRP group had similar or higher ERD than the low CRP group in all entries. In particular, for “it is distressing that I cannot enjoy eating”, “I am concerned that I will become weaker if I cannot eat”, “I have arguments with my family about food”, and “I am troubled that my family seems to try to force me to eat”, the scores for the high CRP group were significantly higher than those for the low CRP group (*p* = 0.048, 0.022, 0.014, and 0.017, respectively). Both total scores of the QERD-P long version and short version were significantly higher in the high CRP group than in the low CRP group (*p* = 0.040 and 0.029, respectively). The high CRP group had significantly higher ERD than the low CRP group. 

Relationships between the serum CRP levels and QOL measured using the EORTC QLQ-C30 are shown in Table 3. Except for appetite loss in the symptom scales, there were no significant differences between the groups for all items.

Results of the multivariate logistic regression analysis that was performed to examine the associations between the serum CRP levels and the ERD assessed using the QERD-P long and short versions are shown in Table 5. The ERD scores were divided into two categories using the medians (80 for the QERD-P long version and 26 for the QERD-P short version). The high CRP group had significantly higher risks for ERD than the low CRP group (odds ratio [OR] 2.13, 95% confidence interval [CI] 1.10–4.11, *p*-value 0.024; OR 2.06, 95% CI 1.05–4.05, *p*-value 0.035, respectively). 

## 4. Discussion

We conducted a survey to investigate when it was appropriate to initiate multimodal interventions provided by a multidisciplinary team. Specifically, a self-reported questionnaire was distributed among patients with advanced cancer referred to palliative care. As a result, there were significant associations between serum CRP levels and dietary intake, as well as associations with some NISs and ERD. A serum CRP value of 5 mg/dL was considered instrumental for identifying patients who should be provided multimodal care for cancer cachexia. 

The prompt provision of multimodal interventions, or situation-specific supportive and palliative care, by a multidisciplinary team from early onset to late cachexia is essential in ideal patient- and family-centered care. However, no standard care has been established to manage patients’ symptoms and the distress experienced by both patients and family caregivers, resulting from cancer cachexia [11,12]. Furthermore, there has been no consensus on when multimodal care for cancer cachexia should start, even though beneficial effects are expected by applying early combined intervention comprising nutritional therapy, exercise, and pharmacotherapy [11,12,13,14,15]. The present study revealed that patients with a serum CRP level of 5 mg/dL or more already had reduced dietary intake, some NISs (e.g., appetite loss and drowsiness), and high ERD. Therefore, this CRP value may be a clinically critical point for the initiation of multimodal care for patients and family caregivers affected by cancer cachexia in supportive and palliative care. 

Studies have suggested that systemic inflammation is implicated in the responsible mechanisms of cancer cachexia [1,2,3,4,5,6,7]. Pro-inflammatory cytokines stimulate the CNS, particularly the hypothalamic–pituitary–adrenal (HPA) axis, that dominates the sympathetic nervous system. Activation of the HPA axis induces the secretion of cortisol and the generation of glucocorticoids in the adrenal gland, resulting in muscle atrophy [8]. Thus, systemic inflammation induces energy imbalances and abnormal protein and lipid metabolism (i.e., hyper-catabolism in proteolytic and lipolytic pathways) and catabolism in muscle and adipose tissue [9]. Moreover, systemic inflammation is correlated with physical and psychological symptoms (e.g., fever, appetite loss, fatigue, drowsiness, anxiety, and cognitive alterations), which may be directly affected by the alterations in the CNS and HPA axis [16,17,18,19,20,21,22,23,24,25]. In turn, these symptoms generate emotional distress, which can promote systemic inflammation and alter the CNS and HPA axis. Physical and psychological symptoms and emotional distress generally co-exist and amplify one another [9]. Moreover, these problems can disrupt cachectic patients’ circadian rhythms and their lives (e.g., eating and sleeping habits) [9]. However, there is insufficient evidence suggesting that systemic inflammation acts through alterations in the CNS and HPA axis in the genesis of physical and psychological symptoms and emotional distress in patients with advanced cancer [8,9,10]. Ultimately, controlling systemic inflammation (i.e., lowering CRP levels) may reduce its impacts on the CNS and HPA axis, alleviate symptoms, and solve the cachexia-related complications. The clinical implication of serum CRP levels in people affected by cancer cachexia needs to be further investigated, considering the impacts of systemic inflammation on the CNS and HPA axis, to improve their disrupted lives. 

This study has several strengths and limitations. Approximately 70% of the patients analyzed in this study were undergoing anticancer treatment in multiple designated cancer hospitals, which means that the results may reflect the actual situation during cancer treatment. However, there are limitations to the generalization of the results obtained from Japanese inpatients. Moreover, serum CRP levels are not a marker specific to cancer cachexia, and they can also be elevated by infection and cancer treatment, including cytotoxic anticancer agents and radiotherapy. Furthermore, only data obtained at the time of the survey were used in this study, although CRP values fluctuate during the clinical course [35]. However, a CRP level of 5 mg/dL in patients with advanced cancer was considered to be effective in the screening of patients and their family caregivers at risk for negative impacts of cancer cachexia. 

We previously reported that the number of NISs was significantly associated with ERD as well as reduced dietary intake in the similar population [36]. However, we have to consider the differences in ERD between sexes and various cancers and the impacts of co-existing autoimmune or inflammatory diseases on the systemic inflammation. Further research is needed to verify the present findings and clarify their relevance in clinical practice. 

## 5. Conclusions

Higher CRP levels were significantly associated with reduced dietary intake, more severe appetite loss, vomiting, and drowsiness, and higher ERD among patients with advanced cancer receiving supportive and palliative care. A serum CRP value of 5 mg/dL may be one indicator used to initiate multimodal care for cancer cachexia. Cachexia-related research needs to refocus on systemic inflammation of what we already know as well as deeper understanding of the mechanisms of cancer cachexia.

## Figures and Tables

**Table 1:** Patient characteristics according to serum CRP levels.

	CRP < 5	CRP ≥ 5	*p* Value
	(n = 117)	(n = 75)	
Age in years	68.0 (56.0, 76.0)	66.0 (51.0, 74.0)	0.102
Sex			0.365
Female	64 (54.7%)	36 (48.0%)	
Male	53 (45.3%)	39 (52.0%)	
Primary cancer site, n (%)			0.117
Upper and lower gastrointestinal tracts	12 (10.3%)	12 (16.0%)	
Liver, biliary system, and pancreas	20 (17.1%)	13 (17.3%)	
Lung	27 (23.1%)	13 (17.3%)	
Breast	9 (7.7%)	3 (4.0%)	
Gynecological	8 (6.8%)	15 (20.0%)	
Urological	9 (7.7%)	5 (6.7%)	
Head and neck	6 (5.1%)	4 (5.3%)	
Hematologic malignancy	11 (9.4%)	2 (2.7%)	
Others	15 (12.8%)	8 (10.7%)	
Treatment status			0.072
Pre-chemotherapy	17 (14.5%)	7 (9.3%)	
Chemotherapy	86 (73.5%)	50 (66.7%)	
Never treated/previous treatment	14 (12.0%)	18 (24.0%)	
ECOG PS, n (%)			0.045
0	2 (1.7%)	0 (0.0%)	
1	28 (23.9%)	9 (12.0%)	
2	44 (37.6%)	23 (30.7%)	
3	37 (31.6%)	38 (50.7%)	
4	6 (5.1%)	5 (6.7%)	
Body mass index (kg/m^2^)	21.3 (18.9, 24.2)	20.2 (18.7, 23.4)	0.464
Weight loss rate over 6 months (%)	5.8 (0.3, 9.9)	7.2 (3.1, 13.8)	0.043
Cachexia/refractory cachexia, yes	71 (60.7%)	52 (69.3%)	0.237
Pleural effusion, ascites, or edema affecting weight, yes	17 (14.5%)	25 (33.3%)	0.003
Albumin (g/dL)	3.5 (3.1, 3.8)	2.8 (2.4, 3.2)	<0.001
CRP (mg/dL)	0.7 (0.2, 2.1)	9.2 (7.0, 12.7)	<0.001

Values represent n (%) or median (interquartile range). CRP, C-reactive protein; ECOG PS, Eastern Cooperative Oncology Group performance status.

**Table 2:** Relationships between serum CRP levels, dietary intake, and nutrition impact symptoms.

	CRP < 5	CRP ≥ 5	*p*-Value
Dietary intake			
Dietary intake score	5.0 (3.0, 7.0)	4.0 (2.0, 5.0)	0.002
Number of patients with a dietary intake score of 7 or less	93 (79.5%)	70 (93.3%)	0.009
Symptoms that interfere with patients’ ability to ingest or digest nutrients			
Oral pain	0.0 (0.0, 1.0)	0.0 (0.0, 1.0)	0.751
Appetite loss	5.0 (1.0, 7.0)	5.0 (3.0, 7.0)	0.304
Early satiety	5.0 (2.0, 7.5)	5.0 (2.0, 8.0)	0.956
Nausea	1.0 (0.0, 3.0)	1.0 (0.0, 3.0)	0.661
Vomiting	0.0 (0.0, 1.0)	0.0 (0.0, 2.0)	0.012
Constipation	3.0 (0.0, 6.0)	5.0 (0.0, 7.0)	0.458
Diarrhea	0.0 (0.0, 3.0)	0.0 (0.0, 5.0)	0.363
Abnormal taste	0.0 (0.0, 3.0)	1.0 (0.0, 7.0)	0.219
Abnormal smell	0.0 (0.0, 2.0)	0.0 (0.0, 1.0)	0.534
Dry mouth	2.0 (0.0, 4.0)	2.0 (0.0, 6.0)	0.108
Dental problems	0.0 (0.0, 2.5)	0.0 (0.0, 2.0)	0.489
Difficulty swallowing	0.0 (0.0, 2.0)	0.0 (0.0, 2.0)	0.625
Food bolus obstruction	0.0 (0.0, 3.0)	0.0 (0.0, 3.0)	0.533
Symptoms that compromise patients’ desire to eat and take nutrients			
Fatigue	3.0 (2.0, 6.0)	4.0 (2.0, 5.0)	0.709
Drowsiness	3.0 (1.0, 6.0)	5.0 (2.0, 6.0)	0.041
Anxiety	2.5 (1.0, 5.0)	3.0 (0.0, 5.0)	0.823
Feeling sad	3.0 (1.0, 5.0)	3.0 (1.0, 5.0)	0.982
Symptoms that indirectly compromise patients’ food and nutrient intake			
Pain other than oral pain	3.5 (0.0, 7.0)	3.0 (0.0, 7.0)	0.738
Shortness of breath	2.0 (0.0, 5.0)	1.0 (0.0, 3.0)	0.494
Number of nutrition impact symptoms with a score of 4 or more	6.0 (2.0, 8.0)	6.0 (4.0, 9.0)	0.212

Values represent n (%) or median (interquartile range). Serum CRP levels were measured in mg/dL. Dietary intake was assessed using the IVVAS (10-point scale). High scores indicate better dietary intake. Symptoms were rated between 0 and 10. High scores indicate worse symptoms. CRP, C-reactive protein; IVVAS, Ingesta-Verbal/Visual Analog Scale.

**Table 3:** Relationships between serum CRP levels and QOL assessed using the EORTC QLQ-C30.

	CRP < 5	CRP ≥ 5	*p*-Value
Functional scales			
Physical functioning	53.3 (33.3, 73.3)	46.7 (26.7, 73.3)	0.122
Role functioning	33.3 (0.0, 66.7)	33.3 (0.0, 66.7)	0.604
Emotional functioning	66.7 (50.0, 83.3)	66.7 (50.0, 83.3)	0.221
Cognitive functioning	66.7 (33.3, 83.3)	66.7 (50.0, 66.7)	0.957
Social functioning	50.0 (33.3, 83.3)	50.0 (33.3, 83.3)	0.728
Symptom scales			
Fatigue	66.7 (44.4, 88.9)	77.8 (55.6, 88.9)	0.067
Nausea and vomiting	16.7 (0.0, 33.3)	16.7 (0.0, 50.0)	0.419
Pain	66.7 (33.3, 100.0)	83.3 (50.0, 100.0)	0.222
Dyspnea	33.3 (33.3, 66.7)	33.3 (0.0, 100.0)	0.869
Sleep disturbance	66.7 (33.3, 100.0)	66.7 (33.3, 100.0)	0.612
Appetite loss	66.7 (33.3, 100.0)	66.7 (33.3, 100.0)	0.008
Constipation	33.3 (0.0, 66.7)	33.3 (33.3, 66.7)	0.158
Diarrhea	0.0 (0.0, 33.3)	33.3 (0.0, 66.7)	0.181
Global health status	33.3 (16.7, 50.0)	16.7 (16.7, 41.7)	0.096
Financial difficulties	33.3 (0.0, 66.7)	33.3 (0.0, 66.7)	0.560

Values represent median (interquartile range). Serum CRP levels were measured in mg/dL. QOL was assessed using the EORTC QLQ-C30. High scores indicate higher QOL in functional scales and global health status or worse symptoms and difficulties in symptom scales and financial difficulties. CRP, C-reactive protein; EORTC QLQ-C30, European Organization for Research and Treatment of Cancer Core Quality of Life Questionnaire; QOL, quality of life.

**Table 4:** Relationships between serum CRP levels and eating-related distress.

	CRP < 5	CRP ≥ 5	*p*-Value
Factor 1: Reduced dietary intake			
**It is distressing that I cannot eat even though I want to eat more.**	5.0 (3.0, 6.0)	5.0 (3.0, 6.0)	0.308
It is distressing that I cannot enjoy eating.	5.0 (3.0, 6.0)	6.0 (4.0, 7.0)	0.048
It is distressing that I get full quickly and cannot eat enough.	4.0 (3.0, 6.0)	5.0 (3.0, 6.0)	0.245
Factor 2: Reasons why I cannot eat			
**I do not understand the reason why I cannot eat.**	3.0 (1.0, 5.0)	3.0 (2.0, 5.0)	0.126
I do not understand the reason why I do not have an appetite.	3.0 (1.0, 4.0)	3.0 (2.0, 5.0)	0.100
I do not understand the reason why I cannot eat enough.	3.0 (1.0, 5.0)	3.0 (2.0, 5.0)	0.121
Factor 3: Becoming weaker			
**I am concerned that I will become weaker if I cannot eat.**	5.0 (3.0, 6.0)	6.0 (5.0, 7.0)	0.022
I am concerned that I will lose muscle strength if I cannot eat.	5.0 (4.0, 6.0)	6.0 (5.0, 7.0)	0.090
I am concerned that I will lose weight if I cannot eat.	5.0 (3.0, 6.0)	5.5 (4.0, 6.5)	0.117
Factor 4: Insufficient information			
**I have insufficient information about which nutrients I should prioritized.**	4.0 (3.0, 6.0)	5.0 (3.0, 5.5)	0.321
I have insufficient information about which nutrients I should avoid.	4.0 (3.0, 5.0)	5.0 (3.0, 5.0)	0.731
I have insufficient information about which nutritional supplements I should take.	4.0 (3.0, 5.0)	5.0 (3.0, 5.0)	0.460
Factor 5: Arguments with my family			
**I have arguments with my family about food.**	2.0 (1.0, 3.0)	2.0 (1.0, 4.5)	0.014
I am troubled that my family seems to try to force me to eat.	1.0 (1.0, 3.0)	2.0 (1.0, 4.0)	0.017
I get frustrated with my family over food.	1.0 (1.0, 3.0)	1.5 (1.0, 4.0)	0.274
Factor 6: Change in appearance			
**It’s hard for me that my appearance had changed a lot from before as I became thin.**	3.0 (1.0, 5.0)	4.0 (2.0, 5.0)	0.157
It’s hard for me to be seen by others as so skinny.	3.0 (1.0, 5.0)	3.0 (2.0, 5.0)	0.635
It’s hard to see myself as so skinny.	3.0 (1.0, 5.0)	4.0 (2.0, 5.0)	0.149
Factor 7: Time with my family			
**I spend less time talking with my family because I do not eat with them.**	3.0 (2.0, 5.0)	4.0 (1.0, 5.0)	0.557
I spend less time enjoying with my family during meals.	4.0 (2.0, 5.0)	4.5 (1.0, 6.0)	0.372
I spend less time in daily life with my family because I cannot eat.	3.0 (1.0, 4.0)	4.0 (1.0, 5.0)	0.281
Total score of the long version	76.0 (58.0, 92.0)	83.0 (66.0, 99.0)	0.040
Total score of the short version	25.0 (20.0, 31.0)	28.0 (23.0, 33.0)	0.029

Values represent median (interquartile range). Serum CRP levels were measured in mg/dL. Eating-related distress was assessed using the Questionnaire for Eating-Related Distress among Patients with advanced cancer (7-point scale). Boldfaced items indicate those belonging to the short version. High scores indicate worse distress. CRP, C-reactive protein.

**Table 5:** Association between serum CRP levels and eating-related distress assessed using the QERD-P (n =181).

	The QERD-P Long Version		The QERD-P Short Version	
	Odds Ratio (95% CI)	*p*-Value	Odds Ratio (95% CI)	*p*-Value
CRP levels				
<5 mg/dL	Reference		Reference	
≥5 mg/dL	2.13 (1.10, 4.11)	0.024	2.06 (1.05, 4.05)	0.035
Age in years	0.99 (0.97, 1.02)	0.640	0.99 (0.97, 1.02)	0.490
Sex				
Female	Reference		Reference	
Male	1.49 (0.78, 2.84)	0.230	1.72 (0.89, 3.33)	0.105
Primary cancer site				
Lung	Reference		Reference	
Gastrointestinal	1.56 (0.63, 3.85)	0.334	1.77 (0.70, 4.47)	0.225
Others	0.77 (0.33, 1.83)	0.559	0.94 (0.39, 2.23)	0.883
Treatment status				
Non-chemotherapy	Reference		Reference	
Chemotherapy	1.01 (0.50, 2.03)	0.974	1.17 (0.58, 2.37)	0.667
ECOG PS				
0, 1	Reference		Reference	
2	0.62 (0.25, 1.51)	0.294	0.84 (0.34, 2.07)	0.706
3, 4	0.73 (0.30, 1.76)	0.484	1.20 (0.49, 2.95)	0.683

Serum CRP levels, age, sex, primary cancer site, treatment status, and ECOG PS were included in the multivariate analysis. CI, confidence interval; CRP, C-reactive protein; ECOG PS, Eastern Cooperative Oncology Group performance status; QERD-P, Questionnaire for Eating-Related Distress among Patients with advanced cancer.

## Data Availability

The original contributions presented in this study are included in the article. Further inquiries can be directed to the corresponding author.
